# Cellular Therapies for the Treatment of Hematological Malignancies; Swine Are an Ideal Preclinical Model

**DOI:** 10.3389/fonc.2019.00418

**Published:** 2019-06-21

**Authors:** Raimon Duran-Struuck, Christene A. Huang, Abraham J. Matar

**Affiliations:** ^1^Department of Pathobiology, University of Pennsylvania School of Veterinary Medicine, Philadelphia, PA, United States; ^2^Department of Surgery, University of Colorado, Denver, CO, United States; ^3^Department of Surgery, Emory University School of Medicine, Atlanta, GA, United States

**Keywords:** miniature swine, lymphoma and leukemia, transplantation, cell therapy, SCID

## Abstract

The absence of clinically relevant large animal tumor models has historically forced experimental cellular therapies for hematological malignancies to translate directly from murine models to clinical trials. However, recent advances highlight swine as an ideal large animal model to demonstrate the safety of murine proof of concept studies prior to their implementation clinically. The availability of the MHC defined MGH miniature swine herd has been key for the development of novel approaches for hematopoietic cell and solid organ transplantation. New spontaneously arising hematological malignancies in these swine, specifically myeloid leukemias and B cell lymphomas, resemble human malignancies, which has allowed for development of immortalized tumor cell lines and has implications for the development of a large animal transplantable tumor model. The novel development of a SCID swine model has further advanced the field of large animal cancer models, allowing for engraftment of human tumor cells in a large animal model. Here, we will highlight the advantages of the swine pre-clinical model for the study of hematological malignancies. Further, we will discuss our experience utilizing spontaneously arising tumors in MGH swine to create a transplantable tumor model, describe the potential of the immunodeficient swine model, and highlight several novel cellular and biological therapies for the treatment of hematological malignancies in swine as a large animal pre-clinical bridge.

## Introduction

Preclinical murine models have long been the foundation for mechanistic studies and assessment of therapeutic strategies for human disease. While the mouse has provided a cheap, reproducible, and easy to use model whose role will never be replaced, the extrapolation of mouse studies directly to clinical application has largely been unsuccessful, especially with respect to cancer (1–3). This is likely due to the vast number of genetic, immunologic, and physiological differences between mice and humans. Murine models often recapitulate a specific pathway within a disease, but frequently do not provide the entire spectrum of physiologic changes that occur in humans, preventing direct translation of therapeutic strategies. Large animals provide a more clinically relevant model to study cancer as they are significantly more similar to humans in terms of anatomy, physiology, genetics, and immunological responses. Some however, may challenge the ethical aspects of using large animals for research purposes. Among the large animals used for pre-clinical research purposes, primates, canines, and swine are the three most common. Primates are most similar to humans with respect to physical and anatomic characteristics (4, 5), and there are an abundance of human reagents with cross-reactivity to primates. However, the use of primates in research is often hindered by strict regulations, potential for communicable diseases, the requirement for significant personnel training and personal protective equipment, societal protest, and expense. Canine and swine models provide a more practical option with respect to ease of breeding and handling, shorter gestation periods, and large litters, while maintaining an anatomy and physiology that is similar to humans (6). To date, there are a limited number of large animal models of hematological malignancies (7–10). However, the existing models, specifically swine models, have demonstrated that large animal hematological malignancies share important similarities to human malignancies (11–13). Further, the swine model is increasingly being used in the setting of anti-cancer drug development (14). Here we will highlight the advantages of the swine pre-clinical model for the study of hematological malignancies, while also reviewing existing swine models and exploring novel therapeutic strategies, both existing and on the horizon (8, 15).

## Swine as a Preclinical Model of Malignancy

**Figure 1:**
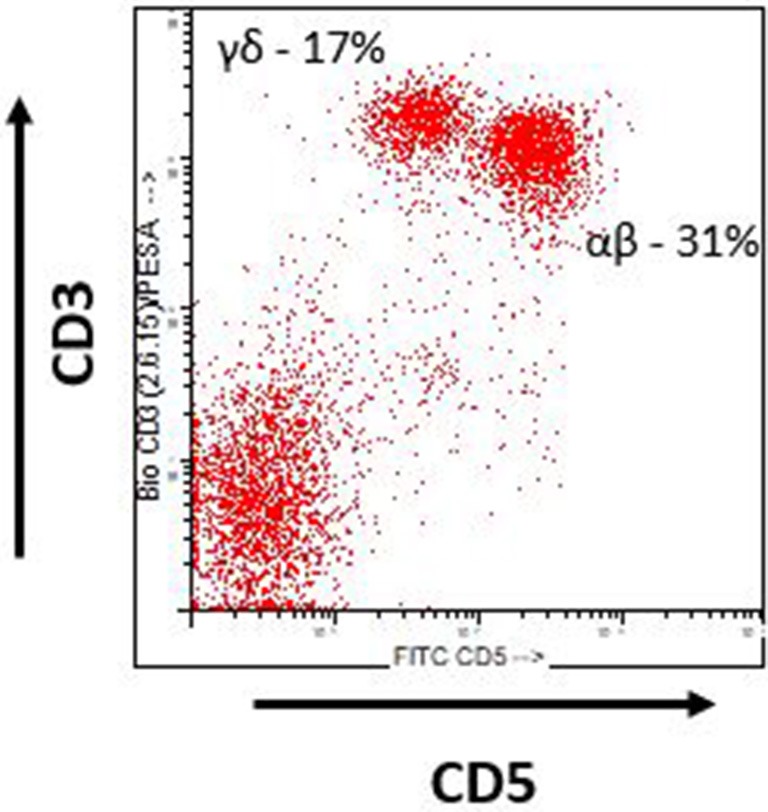
Swine T cells. Flow cytometric analysis of swine lymphocytes from a naïve MGH miniature swine. Alphabeta (αβ) and Gammadelta (γδ) can easily be identified by expression of CD3 and CD5. Alphabeta T cells are CD3+ CD5 hi and Gammadelta T cells are CD3+ CD5 lo.

Swine as a preclinical model of hematologic malignancy offer several advantages over other species one of which is a similar immune profile, specifically the lymphocyte repertoire. Despite the similarities, there are several important differences to note. With respect to T cell populations, both humans and swine possess two distinct lineages of T cells based on the alphabeta or gammadelta T cell receptor (16). Alphabeta T cells in both species recognize foreign antigen in an MHC dependent fashion, while gammadelta T cells recognize foreign antigen in a non-MHC dependent fashion. One major difference however is the fact that swine possess significantly higher numbers of gammadelta T cells than do humans, particularly in the peripheral blood and intestinal lymphoid tissues (16). Experimentally, swine alphabeta and gammadelta T cells can be easily distinguished based on CD3 and CD5 expression utilizing flow cytometry (Figure 1). As a result, swine represent an ideal model for the study of gammadelta T cell responses in the setting of malignancy, a previously underexplored area.

Both swine and humans possess traditional T helper cells, CD4+CD8-, which recognize antigen in a MHC class II dependent manner, and cytolytic T cells, CD4-CD8+ which recognize antigen in a MHC class I dependent manner. Swine however possess significantly more cytolytic CD8+ T cells than T helper CD4+ cells in circulation, which is the opposite of the human T cell repertoire (16). Further, while CD4+CD8+ T cells exclusively reside in the thymus in humans, CD4+CD8+ T cells can be found in extrathymic locations in swine, and are differentiated from CD4+CD8+ thymocytes by expression of CD8 alpha alpha homodimer and lack CD1 expression. Peripheral CD4+CD8^lo^ cells are memory T helper (Th) cells, distinct from naïve Th cells which are CD4+CD8−. These memory Th cells acquire the CD8 alpha alpha homodimers as a result of antigen exposure (16).

Another advantage of the swine model is the abundance of swine specific reagents available. Historically, a barrier in swine research has been the relative lack of swine specific reagents. However, a recent study outlined enormous progress on this front, specifically identifying swine cluster of differentiation (CD) markers and linking them to their human counterparts (17). A broad literature review identified 359 known swine CD markers, with over 800 identified reagents including monoclonal antibodies, polyclonal antibodies, and fusion proteins against 266 swine CD markers. With respect to *in vitro* monitoring of the immune system (e.g., flow cytometry), there are commercially available porcine antibodies directed against every major cell type including porcine T cells, B cells, NK cells, T regulatory cells, myeloid cells, dendritic cells, neutrophils, and others. *In vivo*, there are a host of swine specific reagents as previously mentioned including depleting antibodies targeted against CD3, CD4, CD8, and Tregs (17). The effects of a novel rabbit anti-porcine anti-thymocyte globulin (ATG) have been investigated. Rabbit anti-human ATG is a commonly used agent clinically in the setting of conditioning prior to hematopoietic cell transplantation (HCT), treatment of graft vs. host disease (GVHD), and for treatment of acute cellular rejection after solid organ transplantation. In swine, rabbit anti-porcine ATG is a poor T cell depletion agent (unpublished data). In comparison, two anti-CD3 immunotoxins were superior. The chemically conjugated swine anti-CD3-immunotoxin provides robust T cell depletion in swine (18) while a recombinant (less toxic version) was also relatively effective with a 80% decrease in CD3 T cells in the peripheral blood (19). These findings have been previously documented in other species and humans supporting that monoclonal antibodies are less potent at immunodepletion within tissues when compared to immunotoxins (20–22). There have also been several porcine recombinant fusion toxins generated, specifically a porcine IL-2 fusion toxin for *in vivo* depletion of swine CD25+ cells and a porcine CTLA-4 fusion toxin for depletion of antigen presenting cells (APCs) (23). There is also evidence that human therapeutics can cross react with corresponding porcine targets *in vivo* with great efficacy (24).

Finally, given the recent shift in treatment of cancer toward immunotherapies, there is a growing need for new biomarkers that are predictive for treatment stratification, monitoring and response. Swine are an ideal model for the discovery and validation of novel biomarkers given their physiologic and immune similarities to humans as described previously. Advantages include the ability to longitudinally follow swine over a period of years given their long life span and the relative ease in obtaining large quantities of blood, serum, and tissue samples (25). Importantly, existing swine models of cancer have demonstrated similarities in biomarkers compared to their human counterparts. In an oncopig model of hepatocellular carcinoma (HCC), alpha feto protein (AFP) was reliably used for detection of swine HCC as well as treatment monitoring (26). With respect to hemolymphatic malignancies, in swine PTLD, LDH is a reliable marker of hemolysis and tumor development (13). The development of reliable swine models of hemolymphatic malignancies has enormous potential to uncover novel biomarkers.

## Large Animal Models of Lymphohematopoietic Malignancies

**Figure 2:**
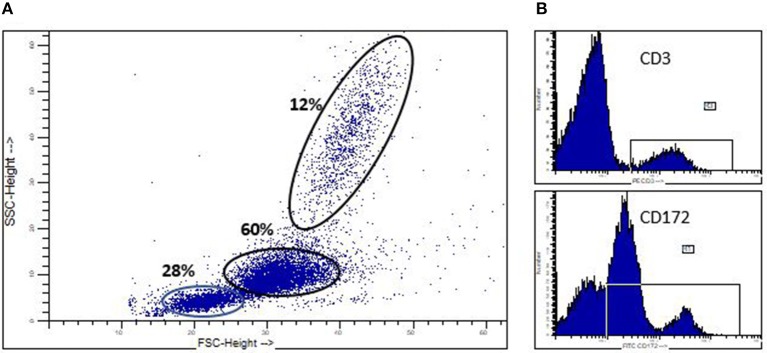
Spontaneoulsy arising swine CML. **(A)** Flow cytometric analysis of peripheral blood in a swine which spontaneously developed CML, demonstrating a significant myeloblastosis. **(B)** CD3+ and CD172+ staining demonstrating the malignancy is of myeloid origin (CD172+) and not lymphocyte origin (CD3+).

We previously reported our identification of spontaneously developing chronic myelogenous leukemia (CML) in the Massachusetts General Hospital (MGH) major histocompatibility complex (MHC) defined miniature swine herd (11). Through years of selective breeding, the MHC genes of these swine have been “fixed,” while minor antigens remain variable, thereby providing a valuable large animal model to study transplantation. The CML that spontaneously develops in these swine (sCML) closely resembles human CML (hCML) as confirmed by flow cytometric analysis of peripheral blood mononuclear cells (PBMCs), lymph nodes (LNs), as well as histological examination of tissues obtained at necropsy (11) (Figure 2). The development of hCML is closely associated with a chromosomal translocation *t*_(9, 22)_, also known as the Philadelphia chromosome (Ph+) in over 95% of cases. sCML cell lines isolated from MGH miniature swine were karyotyped to evaluate for an analogous chromosomal translocation. Although the direct translation of a *t*_(9:22)_ translocation could not be made due to disparities in chromosome numbers (23 pairs in humans vs. 19 pairs in swine), a shortened chromosome arm was found, indicating that the development of sCML is likely associated with a chromosomal abnormality. Interestingly, sCML was associated with defects in a nucleoporin gene (Nup107). Defects in this gene have also been associated with human leukemias (27, 28).

The identification of a severe combined immunodeficiency (SCID) pig at Iowa State University has offered a potentially valuable model for the study of hematological malignancies (29). These naturally occurring SCID pigs were found to have two causative mutations in the Artemis gene, a well-characterized gene in human SCID patients (30). SCID pigs share a similar immune profile to that of human SCID patients as they are completely deficient in T and B cells and are thus incapable of producing antibodies or mounting T cell responses. Similar to humans, the SCID pig does have macrophages and natural killer (NK) cells, the latter of which are primarily responsible for the immune response in these animals. The success of engraftment of human hematopoietic stem cells in xenotransplantation studies in mice relies, in part, on the ability of polymorphisms within the murine non-obese diabetic (NOD) signal regulatory protein alpha (SIRPA) gene that dictate its capability to be activated by human CD47 (31) on hematopietic cells. Signaling of these two molecules confers phagocytic tolerance to human stem cells by the murine monocytic/macrophage innate immune arm. Interestingly, macrophages from SCID pigs did not reject human lymphohematopoietic cells, thus demonstrating a NOD phenotype (32). However, xenogeneic tumor studies in which human pancreatic and melanoma cell lines were introduced into SCID pigs revealed an NK cell infiltrate in tumors in a subset of pigs. Despite this NK cell infiltrate, these pigs did not reject the xenografts (33). The authors hypothesized that the lack of rejection in the setting of an NK cell response was secondary to a deficiency in cytokine production, namely IL-2. Finally, Powell et al. recently reported the development of a spontaneous host-derived T cell lymphoma and a chronic lymphocytic leukemia (CLL) following bone marrow transplantation (BMT) in two SCID pigs (34). The development of a host-derived malignancy following BMT may be related to a “leaky” Artemis gene that allows for generation of lymphocytes, albeit at reduced numbers, as previously documented in human SCID patients (35). Moving forward, whether malignancies arise spontaneously or are introduced from allogeneic or xenogeneic origins, it is clear that the SCID pig will become a potent tool for studying lymphohematopoietic malignancies. Moreover, as the first large animal model to allow for engraftment of human cancer cell lines without concern for rejection, the SCID pig will be invaluable for testing of novel cellular and pharmacological therapies.

**Figure 3:**
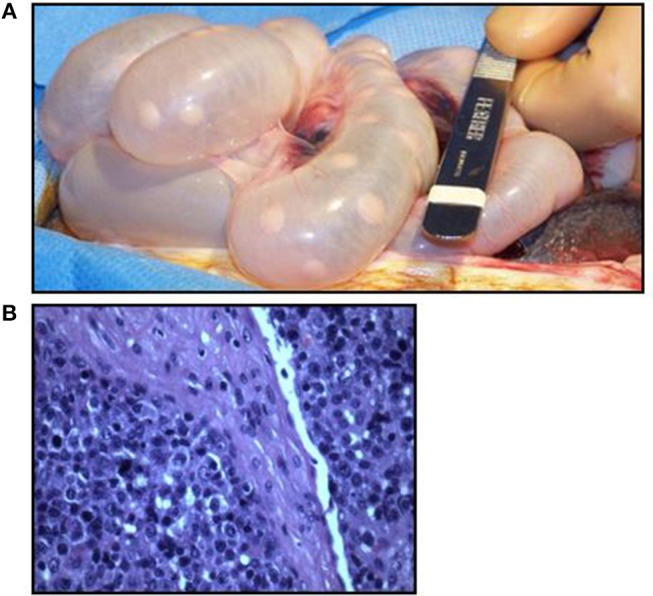
Swine PTLD. **(A)** Significant intestinal lymphadenopathy in a swine with PTLD. **(B)** Histologic analysis of a swine intestinal lymph node demonstrating an acute lymphoblastic process in the setting of PTLD.

Previously we reported the use of the MGH miniature swine as a potential model of B cell lymphomas, specifically post-transplant lymphoproliferative disease (PTLD), which is a potentially lethal complication following transplantation (12, 36). In our experience with both hematopoietic cell transplantation (HCT) and solid organ transplantation (SOT), MGH miniature swine develop PTLD as a result of uncontrolled herpes viruses, either from primary infection or reactivation of a gammaherpesvirus, porcine lymphotropic herpesvirus-1 (PLHV-1) (36) (Figures 3A,B). Clinically, herpes induced lymphomas (HILs) are observed in immunosuppressed patients, such as those with HIV or transplant patients. However, in humans, PTLD is driven by primary infection or reactivation of Epstein Barr virus (EBV) (13) as a result of loss of antiviral function of CD8+ cytotoxic T cells in the setting of immunosuppression. Unfortunately, there is currently no animal model that accurately recapitulates EBV-induced PTLD. To date, rodent models continue to be the most utilized when studying PTLD, with novel therapies being tested in murine xenogeneic models using human PTLDs (37). However, these rodent models cannot accurately replicate potential complications due to their small size (38). Other studies using murine gammaherpesvirus have struggled with their inaccuracy modeling human disease (38). In future studies, the SCID pig may provide an exciting model in which to study EBV driven PTLD in an animal of human size and physiology. It is important to mention that the model is not devoid of limitations. Besides the restrictions of working across xenogeneic barriers, the inherent fragility of SCID swine [which are highly susceptible to infections (35)] and the requirement of housing with room-sized specialized (Biobubbles) and hepa-filtered (ABSL-3 like) animal facilities may prove difficult to many due to expense. However, for the first time, this model will allow for the assessment of novel human derived cellular therapies and pharmacological approaches to address PTLD in a large animal model.

## Swine as a Pre-clinical Model to Test Biological and Cellular Therapies

Cellular therapies such as blood transfusions have been used in medicine for decades. One of the most sophisticated cellular therapies—bone marrow transplantation (BMT)—has evolved dramatically since its inception in 1956 and is now used clinically to treat a variety of hematological malignancies and blood dyscracias (39). During BMT, the recipient's immune system is (partly or fully) destroyed by radiation or chemotherapy and replaced by either autologous or allogeneic bone marrow. Unfortunately, frequent complications of allogeneic bone marrow transplantation are infection and graft-vs.-host disease (GvHD), during which donor immune cells attack recipient cells. Swine (40–42), as well as other animal models (43, 44), have played an important role in the study and development of BMT and other cellular therapies for clinical application. Swine are an attractive model for the study and development of cellular immunotherapies due to the abundance of swine-specific reagents available (45–52). We have previously demonstrated the applicability of swine as a clinically-relevant model for the use of cellular therapies in inducing immunological tolerance via mixed chimerism (in which both recipient and donor cells co-exist) as well as for the treatment of GvHD in the form of donor leukocyte infusions (DLIs) (42, 53). In the following, we will recap some of these studies and touch on novel cellular therapies on the horizon.

### Donor Leukocyte Infusions (DLI)

The use of donor leukocyte infusions (DLI) following allogeneic HCT to both augment anti-tumor responses and enhance immune cell engraftment has expanded dramatically since its introduction 30 years ago (54). DLIs utilize donor peripheral blood leukocytes collected via apheresis, a process in which blood components are separated via density gradient and lymphocytes and monocytes are harvested, while granulocytes are returned to the patient. DLI following HCT in the setting of hematological malignancy has several indications, including as a prophylactic therapy for patients with a high risk of relapse, treatment of PTLD and viral infections and as a rescue therapy for those with graft failure. However, GVHD remains one of the most feared side effects of allogeneic BMT. Important studies by Sachs' group demonstrated the value of swine as a cell therapy model to optimize and harness the anti-leukemia effects of allogeneic HCT while avoiding GvHD. These studies in swine exploited a novel HCT approach first shown in mice in which the establishment of mixed chimerism across MHC barriers promoted immune tolerance, thus preventing GvHD (55, 56). This study and other similar studies in non-human primates laid the foundation for Sachs' pioneering study in humans using allogeneic BMT to simultaneously treat multiple myeloma and induce immune tolerance to MHC mismatched kidney allografts (57). Using the swine model described above, we attempted to leverage the alloreactive properties of DLIs to enhance donor chimerism, thereby maintaining or preventing graft loss, while at the same time avoiding GvHD (42). In our study of a total of 33 clinically dosed DLIs infused to immune tolerant swine chimeras, 21 failed to induce conversion to full donor hematopoietic chimerism or cause GvHD, demonstrating that our reduced intensity conditioning regimen for HCT promotes mixed chimerism and an immune tolerant state that is strongly resistant to DLI and GvHD. In several animals, we were able to demonstrate that DLI mediated GvH reactivity could be overcome by significantly increasing the DLI dose, removing chimeric host peripheral blood cell populations (thought to be regulatory T cells) through extensive leukapheresis of the recipient immediately prior to DLI, or delivering lymphocytes fully mismatched to host MHC, but not to donor MHC. However, conversion to full donor chimerism in these scenarios was often associated with severe GvHD, highlighting the importance of mixed chimerism for maintaining immune tolerance. More refined DLIs with selected effector populations are currently being developed and swine will play an important role in determining their efficacy.

### γδT Cells Infusions

γδT cells are a conserved subset of T cells with a distinct surface receptor and which mediate innate immune responses and promote immune surveillance (58). The abundance of γδT cells in humans ranges from 1 to 20% in the peripheral blood and constitutes the major cell population in skin and mucosa (59). Swine γδT cells have been previously characterized and can be tracked in the peripheral blood and tissues using the monoclonal antibody PPT27 (58, 59). γδT cells play a known role in the anti-tumor response and can therefore potentially be used as a potent cellular therapeutic in the context of BMT. However, infusion of donor type γδT cells in a murine model of acute GVHD (aGHVD) mice increased the severity of aGVHD while the absence of host type γδT cells was associated with reduced antigen presenting cell (APC) activation and aGVHD in an MHC-mismatched model (59). By contrast, aGVHD severity was not altered in a MHC-matched, minor antigen (miHA) disparate model of HCT (58, 60). Studying the role of γδT cells in the setting of MHC disparity using a large animal model may prove useful amidst growing evidence of the immune modulatory effects of these cells.

γδT cells provide potent anti-tumor responses to both solid and hematopoietic malignancies, including lymphoma and multiple myeloma (61). As opposed to αβT cells, γδT cells are not MHC restricted when it comes to antigen recognition and do not require APCs for processing immunogenic peptides, allowing them to quickly reactivate during an immune response. Interestingly γδT cells can be activated and expanded *in vivo* through the use of bisphosphonates, which inhibit farnesyl pyrophosphate synthase (62). Taken together, the unique immune properties of γδT cells, as well as their ability to be activated *in vivo* using conventional drugs, make them an attractive option for experimental use in swine models of HCT. Thus far, there have been limited studies utilizing γδT cells in preclinical models or clinical settings. Using a large animal preclinical model such as swine, where γδT cells have been well-characterized, could allow for optimization of important parameters including dosing, route (systemic vs. intratumoral), kinetics, and *ex-vivo* manipulation. More importantly, safety studies in an outbred swine will help discern the conflicting γδ T cell GVHD murine studies and facilitate the expanded use of γδT cells in clinical settings.

### NK Cell Therapies

NK cells are a type of innate lymphoid cell that mount allogeneic immune responses in a non-MHC restricted manner. NK cells distinguish “self” vs. “non-self” through interactions between the killer inhibitor receptor (KIR) expressed on their surface and HLA class I expressed on the surface of host tissues. Recognition of “self” HLA class I by the KIR results in an inhibitory signal, while the absence of HLA class I expression stimulates NK cell activation. NK cells play a pivotal role in the anti-tumor response in cancer cells that down-regulate HLA expression to escape recognition by T cells.

Clinically, NK cells provide a powerful anti-leukemia effect in the setting of allogeneic HCT. For the treatment of acute myelogenous leukemia (AML), donor NK cell alloreactivity from KIR mismatched donors displayed an anti-leukemia effect as part of T cell depleted grafts, while simultaneously providing protection against GVHD (63). Based on these anti-leukemic effects in the absence of GVHD, adoptive NK cell therapy has also been studied as an alternative to unmanipulated DLI for leukemic relapse. NK-DLI was demonstrated to be both a feasible and safe option in a study of 30 patients receiving nonmyeloblative allogeneic stem cell transfer (SCT). CD56+ selected NK cells were given as an NK-DLI 8 weeks after initial transplant. Patients tolerated the DLI well without significant GVHD (64). In the non-transplant setting, administration of KIR mismatched NK cells in 10 pediatric patients with AML who had achieved complete remission following chemotherapy resulted in transient engraftment and excellent two year overall survival (100%) (65). Studies are also underway to evaluate the potential for activated NK cell therapy in the setting of refractory lymphoma (66).

The recent development of the SCID pig provides an interesting avenue for studying the role of NK cells in both allogeneic and xenogeneic anti-tumor responses in a large animal model (67). Similarly to human NK cells, swine NK cells can be identified by expression of CD3^−^CD16^+^CD56^+^ surface markers (68). Powell et al. demonstrated that the number of NK cells in SCID pigs is approximately eight times higher than in non-SCID pigs and that these NK cells are intrinsically functional, as demonstrated by their ability to be activated *in vitro* and lyse tumor cells at the same rate as NK cells from non-SCID pigs (69). In the absence of circulating T and B cells, methods to activate and harness NK cell immune responses can be specifically evaluated in SCID pigs. For example, as IL-2 can stimulate NK cells against ovarian cancer in a murine model (70), further evaluation of IL-2 and other therapies in the SCID pig may further delineate the clinical relevance of NK cells in anti-leukemia and anti-lymphoma immune responses.

### Chimeric Antigen Receptor (CAR) Cells

CARs are genetically engineered receptors that reprogram T cells to target specific cell surface antigens without the need for MHC interaction. CAR T cell therapy has revolutionized cancer research through the re-direction of T cells to target surface receptors expressed by tumor cells, most notably CD19 which is expressed on B cell malignancies (71). CAR T cells were first shown to have anti-tumor responses in mice and then in humans with refractory hematological malignancies (71–73). Currently, groups are designing novel CARs for applications other than cancer, such as autoimmunity and infectious disease (74). As a result, there is an increased need to test the safety of many of these. Indeed, fatal side-effects have been observed in clinical trials, which argues for the need for improved safety testing prior to clinical application, ideally in large animal models (75).

Given the availability of swine models of B cell lymphomas and myeloid leukemias and the identification of the SCID pig, swine may provide an ideal large animal model for the testing of CAR therapies. Though further refinement of these models is necessary, their use should be encouraged for safety assessments in pre-clinical studies. Because CARs rely on the insertion of gene sequences coding for a monoclonal antibody (with a given antigen specificity) as part of the receptor, a potential limitation of swine models is that the CAR may not recognize the target antigen on swine cells. Though this is a potential limitation, for some constructs with conserved antigens, CARs can be very informative for “off-target effects” (76). Swine may be particularly valuable for assessing the relative kinetics and persistence of individual CARs, as it was recently shown that 4-1BB CARs are longer lived when compared to CD28 CARs in humans (77). The potential use of SCID pigs engrafted with human leukemias/lymphomas to assess cytotoxicity/clearance, dosing, imaging, CAR surveillance and different systemic/local delivery methods may be revolutionary in a field that has been mostly limited to murine models. Furthermore, swine could also be used to test the efficacy of anti- PTLD therapies by either CARs directed to PLHV-1 in the MGH swine or to EBV CARs in the case of humanized swine. The possibilities are endless depending on the approach and research questions asked, and highlight the potential role of swine as a critical player in the pre-clinical space of cellular immunotherapies.

### Immune Checkpoint Blockade

Activation of the host immune system against invading tumor cells has long been the goal of cancer therapeutics. A major breakthrough in this endeavor was the discovery of immune checkpoint proteins, which serve to downregulate the immune response. The first immune checkpoint protein to be well-characterized was cytotoxic T-lymphocyte-associated protein 4 (CTLA4), a receptor found on the surface of regulatory T cells and activated T cells. When CTLA4 is bound to its ligands, CD80 and CD86, on the surface of APCs, it provides an inhibitory signal to the T cell. However, the activation molecule CD28 also binds to CD80/CD86, albeit with a reduced affinity as compared to CTLA4. Thus, in the setting of solid organ transplantation where the goal is to suppress the host immune response to the allogeneic graft, it was hypothesized that CTLA-4 would block CD28 interactions with CD80/86, thereby preventing T cell activation. In support, Belatacept, a novel CTLA4 Ig fusion protein, is an effective form of immunosuppression in organ transplantation in both large animal models and humans (78, 79). Naturally, in the setting of cancer, blocking the interaction between CTLA4 and CD80/86 would serve to activate circulating T cells and theoretically fight off invading tumor cells. Ipilumamb, a monoclonal antibody directed against CTLA-4, was first approved by the FDA in 2011 for the treatment of metastatic melanoma and has provided excellent results (80).

The porcine version of CTLA-4 (pCTLA4) exists in several forms and can suppress human CD4+ T cell responses costimulated by porcine B7. Utilizing a novel diptheria toxin (DT) based recombinant pCTLA4 fusion toxin, Peraino et al. demonstrated effective binding to CD80 expressing porcine cells and subsequent inhibition of protein synthesis in those cells. Follow up studies in mice inoculated with a CD80+ porcine lymphoma cell line showed that mice injected with the DT based pCTLA4 fusion toxin experienced prolonged survival compared to untreated mice. It remains to be seen whether the use of pCTLA-IT has similar effects in swine as what has been observed in murine studies where blocking or removing (genetically) host APCs diminished GVHD by limiting the direct activation of alloreactive T cells (81). In summary, the demonstrated ability of a DT based pCTLA4 to inhibit growth of porcine lymphoma cells provides a foundation for future work in targeting CTLA4 in large animal models of lymphohematopoietic malignancies.

## Conclusions

Cancer research is currently being revolutionized by the development of novel cellular and genetic therapies. Historically, these strategies required testing in small animals due to the absence of reliable large animal cancer models. However, recent advancements in swine including the development of immortalized myeloid and lymphoma cell lines from inbred MHC characterized swine, the accessibility of genetically engineered oncogenic swine (known as onco-pigs and addressed in a companion review in this series), and the ability of engrafting human tumors in the SCID pig, highlight swine as an ideal model for large animal tumor studies.

## Author Contributions

RD-S: Lead the topic. CH: Provided figures and edited the paper with modifications. RD-S and AM: Wrote the manuscript.

### Conflict of Interest Statement

The authors declare that the research was conducted in the absence of any commercial or financial relationships that could be construed as a potential conflict of interest.
